# ZnO Nanoparticles Enhance the Biocontrol Efficacy of *Bacillus siamensis* YZUS007 Against Sclerotinia Stem Rot of *Brassica napus*

**DOI:** 10.3390/jof12090633

**Published:** 2026-08-24

**Authors:** Jianchao Hu, Jianwei Jiang, Guanze Yang, Haodong Wang, Jialong Chen, Juan Gan, Jiayi Wang, Zhengxiang Sun

**Affiliations:** College of Agriculture, Yangtze University, Jingzhou 434025, China; 15067920433@126.com (J.H.); 15572103963@163.com (J.J.); 13190425053@163.com (G.Y.); whd1605@163.com (H.W.); 15973743007@163.com (J.C.); gj13595777617@163.com (J.G.); 13264675061@163.com (J.W.)

**Keywords:** *Sclerotinia sclerotiorum*, *Bacillus siamensis*, zinc oxide nanoparticles, biological control, *Brassica napus*

## Abstract

*Sclerotinia sclerotiorum* causes destructive sclerotinia stem rot and severe yield losses in global rapeseed production. The *Bacillus* biocontrol strain YZUS007 serves as an eco-friendly alternative to synthetic fungicides, yet its control efficacy fluctuates under complex field conditions. This study aimed to investigate whether low-dose ZnO NPs could enhance the biocontrol efficacy of YZUS007 and to elucidate the underlying mechanisms. We isolated and identified the antagonistic strain *Bacillus siamensis* YZUS007 from the rhizosphere soil of healthy *Brassica napus*, which exhibited an in vitro pathogen inhibition rate of 78.4 ± 0.7%. Pot tests showed that YZUS007 (BC) achieved 60.79% control efficacy, whereas the YZUS007 + 20 μg/mL ZnO NPs group (BZ) reached 74.68%. Cell-free filtrate of YZUS007 (EC) had a 50.44% control efficacy, and filtrate with ZnO NPs (EZ) increased efficacy to 58.08%. LC-MS/MS identified four extracellular metabolites with increased relative abundances: cinnamic acid, sesamol, patchouli alcohol and 2-phenylbutyric acid. The combined treatment boosted defense enzyme activities and induced higher expression of the defense-related genes *RBOH*, *PR1* and *WRKY33*. Collectively, low-concentration ZnO NPs significantly enhanced the biocontrol performance of YZUS007 by promoting antifungal metabolite accumulation, providing a low-dose, nanomaterial-assisted strategy for sustainable sclerotinia rot management.

## 1. Introduction

*Brassica napus* L., commonly referred to as oilseed rape, ranks among the world’s staple oil-bearing crops and contributes roughly 13% to the total global output of plant-derived edible oil [1]. Sclerotinia stem rot, a destructive plant disorder induced by the necrotrophic pathogenic fungus *Sclerotinia sclerotiorum* (Lib.) de Bary, constantly restricts oilseed rape yield potential. This pathogen boasts an extraordinarily broad host spectrum, capable of infecting over 400 plant species distributed across 75 distinct botanical families. When severe disease outbreaks take place, farmers may face 10–80% yield reduction alongside remarkable deterioration in seed oil quality [2]. Synthetic fungicides remain the dominant intervention to curb sclerotinia stem rot, mainly on account of their rapid onset and stable disease inhibition effect [3,4]. Nevertheless, long-term over-reliance on these chemical agents has accelerated the emergence of fungicide-resistant pathogen populations. This practice also raises widespread public concerns over agricultural environmental pollution, pesticide residues within edible crop products, and the disruption of native soil microbial communities [5,6,7]. Such cumulative drawbacks have driven researchers to explore more eco-friendly, durable disease control schemes.

Microbe-mediated biological control represents a viable replacement for synthetic fungicides, with merits including minimal ecological footprint, high host–pathogen targeting specificity, and the capacity to trigger systemic resistance inside host plant tissues [8]. Among all available biocontrol microbial resources, species belonging to the genus *Bacillus* have attracted extensive research attention. Their competitive advantages include broad-spectrum antifungal activity, environmental tolerance, and endospore formation [9,10]. As one representative member of this genus, *Bacillus siamensis* has displayed promising biocontrol efficacy against multiple economically harmful phytopathogens, such as *S. sclerotiorum*, *Botrytis cinerea*, and *Fusarium oxysporum*. *Bacillus*-based biocontrol can also be applied to aboveground plant tissues, where bacterial cells and their metabolites may directly interact with foliar pathogens and host defense responses [11,12,13]. *Bacillus* spp. suppress pathogens through antifungal metabolite production, competition, direct pathogen interactions, and activation of plant defense pathways [14,15]. Despite these prominent strengths, field application performance of single *Bacillus* strain biocontrol agents often fluctuates drastically. This instability primarily stems from insufficient synthesis of antagonistic metabolites under open-field conditions and strain vulnerability to temperature, air humidity and soil pH shifts [16].

Advancements in nanotechnology have opened new avenues for plant disease management [17]. Among agricultural nanomaterials, zinc oxide nanoparticles (ZnO NPs) have attracted attention because of their low cost, broad-spectrum bioactivity, and potential nutritional benefits as a zinc source for plants [18,19]. ZnO NPs can suppress phytopathogenic fungi through mechanisms including intracellular reactive oxygen species (ROS) accumulation, disruption of fungal cell membranes, and interference with key metabolic enzymes [20]. Previous studies have mainly focused on relatively high ZnO NP concentrations (50–200 μg/mL) for direct pathogen suppression or combined application with chemical fungicides [21].

Such applications may increase input costs and pose risks to non-target organisms and beneficial microorganisms. Extensive research has characterized the direct antifungal activity of ZnO NPs, whereas their potential as low-dose biostimulants for beneficial bacteria and plants remains less explored [22]. Importantly, Ravi et al. reported that ZnO NP supplementation enhanced the production of surfactin and iturin by an endophytic *Bacillus* strain and was accompanied by improved antifungal activity [23]. This finding suggests that ZnO NPs may enhance bacterial antagonistic capacity by modulating bioactive metabolite production. However, the responses of beneficial bacteria to ZnO NPs are not uniform and may depend on nanoparticle properties, concentration, and exposure conditions. Ahmed et al. reported that nanoparticles including ZnO could affect beneficial soil bacteria by altering cell morphology, membrane integrity, respiration, biofilm formation, and bioactive molecule production [24]. Matyszczuk and Krzepilko investigated sublethal ZnO NP exposure in *Bacillus thuringiensis* and *Bacillus megaterium* and demonstrated that ZnO–bacterium interactions were associated with physiological and metabolic responses [25]. Similarly, ZnO NPs were reported to affect the growth and biofilm formation of *Bacillus subtilis* in a concentration-dependent manner [26]. Rutherford et al. further reported growth-inhibitory effects of ZnO-based particles on both Gram-positive and Gram-negative bacteria [27]. Together, these studies indicate that the effects of ZnO NPs on beneficial microorganisms are concentration- and system-dependent, highlighting the importance of identifying doses that enhance bacterial function without causing substantial inhibition. In addition, recent evidence indicates that ZnO NPs may also influence plant defense directly: In wheat, ZnO NP priming enhanced defense responses and resistance to spot blotch, with 15 mg/L showing the strongest effects [28]. A recent study further reported that combining *Bacillus* culture filtrate with ZnO NPs enhanced disease suppression against Pantoea leaf spot in cucumber, providing additional evidence for the potential of nanoparticle-assisted microbial biocontrol [29]. These findings provide a basis for investigating whether low-dose ZnO NPs can enhance the biocontrol performance of YZUS007 through coordinated effects on bacterial antagonism and plant defense responses. An important unresolved question is why synergistic disease suppression detected on intact plant tissues (*in planta*) is consistently far more remarkable than results acquired via isolated laboratory culture (in vitro).

To address the aforementioned gaps, the present study pursues four interrelated aims: (1) isolate and identify bacterial strains with strong antagonistic activity against *S. sclerotiorum* from the rhizosphere soil of healthy *B. napus* plants; (2) screen a biocompatible ZnO NP concentration that promotes rather than inhibits the target strain’s growth; (3) compare the combined disease-suppression efficiency of ZnO NPs and the screened strain via both in vitro and *in planta* testing systems; and (4) dissect the underlying synergistic mechanisms through targeted metabolite profiling, cell-free filtrate testing, and comprehensive plant defense response detection.

## 2. Materials and Methods

### 2.1. Isolation, Identification and Antagonistic Activity of Strain YZUS007

Rhizosphere soil samples were collected from healthy *Brassica napus* plants in Jingzhou, Hubei Province, China, and 126 bacterial isolates were separated via gradient dilution and spreading on LB agar medium. The *S. sclerotiorum* strain used in this study was obtained from the Fungal Specimen Room at Yangtze University. The dual-culture assay on PDA plates was adopted for primary antagonism screening. A 5-mm mycelial plug of *Sclerotinia sclerotiorum* pre-incubated at 22 °C for 3 days was placed at the plate center, and bacterial isolates were streaked symmetrically on two sides of the plug. After 48 h incubation at 22 °C, the diameters of fungal colonies were recorded to calculate the inhibition rate by the formula below: Inhibition rate (%) = [(Control colony diameter − Treatment colony diameter)/Control colony diameter] × 100.

Strain YZUS007, which exhibited the strongest antifungal capacity in preliminary screening, was selected for subsequent tests. A ZEISS Sigma 360 scanning electron microscope (ZEISS, Oberkochen, Germany; Science Compass Testing Center) was utilized to observe cell morphology, confirming its typical rod-shaped cell characteristics. Strain YZUS007, which exhibited the strongest antifungal capacity in preliminary screening, was selected for subsequent tests. Taxonomic identification was based on partial 16S rRNA and *gyrB* gene sequences. Genomic DNA was extracted using a commercial kit (Tiangen Biotech Co., Ltd., Beijing, China), and the purified PCR amplicons were subjected to bidirectional sequencing by Tsingke Biotechnology Co., Ltd. (Wuhan, China). Sequence similarity was assessed by BLASTn against the NCBI GenBank database (http://www.ncbi.nlm.nih.gov/BLAST, accessed on 25 April 2026), and separate phylogenetic trees were constructed for the 16S rRNA and *gyrB* sequences using MEGA 12 (Molecular Evolutionary Genetics Analysis, Version 12, Temple University, Philadelphia, PA, USA).

Co-cultivation of YZUS007 and *S. sclerotiorum* was carried out to observe morphological variations in pathogen hyphae. A Nikon Eclipse Ni-U light microscope (Nikon, Tokyo, Japan) captured the deformity of hyphal structures. After fixation and dehydration pretreatment, the same SEM equipment was applied to visualize ultrastructural damage on fungal hyphae. A Nikon Eclipse Ni-U fluorescence imaging system was further adopted for dual-staining assays: 2′,7′-dichlorodihydrofluorescein diacetate (DCFH-DA) tracked intracellular reactive oxygen species (ROS) accumulation, while propidium iodide (PI) staining reflected the integrity status of cell membranes.

### 2.2. Characterization of ZnO NPs and Biocompatibility Assay

Zinc oxide nanoparticles (ZnO NPs; catalog number Z820772, average particle size = 30 nm, purity ≥ 99%) were purchased from Macklin Biochemical Co., Ltd. (Shanghai, China). Four complementary techniques, including Fourier-transform infrared spectroscopy (FTIR), UV–Vis spectroscopy, X-ray diffraction (XRD), and transmission electron microscopy (TEM), were used to characterize the physicochemical properties of the ZnO NPs. All characterization services were provided by E-Testing Technology Co., Ltd. (Shanghai, China). Plate colony counting served as the evaluation method to verify the biocompatibility between ZnO NPs and YZUS007. Bacterial suspension with an initial concentration of 1 × 10^8^ CFU/mL was serially diluted 10^6^-fold for colony counting. A total volume of 100 μL diluted suspension was spread onto LB agar plates supplemented with ZnO NPs at gradients of 0, 20, 50 and 100 μg/mL. After 24 h cultivation at 30 °C, colony quantities of each group were counted, with three biological replicates set for all treatments.

### 2.3. Synergistic Antifungal Activity and Mechanism Analysis

#### 2.3.1. Direct Antifungal Activity of ZnO NPs

PDA medium supplemented with serial concentrations of ZnO NPs (0, 20, 50, 100, 200 μg/mL) was prepared to test the independent inhibitory effect on *S. sclerotiorum*. A 5-mm pathogen mycelial plug was placed on the central area of each plate, and mycelial diameters were measured after 48 h incubation at 22 °C.

#### 2.3.2. Synergistic Antifungal Activity via Oxford Cup Method

The Oxford cup assay was implemented to quantify in vitro synergistic antifungal performance. A 5-mm *S. sclerotiorum* mycelial plug was positioned at the center of each PDA plate, and Oxford cups were placed at equal distances surrounding the plug. A volume of 100 μL test solution was added into each cup. Two treatment groups were set for comparison: YZUS007 bacterial culture (BC) and YZUS007 bacterial culture amended with 20 μg/mL ZnO NPs (BZ). Inhibition zone widths were measured after 48 h incubation at 22 °C to calculate antifungal inhibition rates.

#### 2.3.3. LC–MS/MS Profiling of Extracellular Metabolites

Supernatants harvested from 48 h cultures of YZUS007 with or without 20 μg/mL ZnO NPs were first centrifuged at 8000× *g* for 10 min and filtered through sterile filter paper to remove bacterial pellets prior to metabolite extraction. Three rounds of liquid–liquid extraction were performed with an equal volume of ethyl acetate. Combined organic phases were evaporated to dryness, reconstituted with LC-MS-grade methanol, and filtered through a 0.22 μm membrane before instrumental detection. Metabolite profiling was completed on a SCIEX TripleTOF LC-MS/MS platform. Chromatographic separation was achieved on a Kinetex^®^ F5 column (100 mm × 2.1 mm, particle size = 2.6 μm, Phenomenex, Torrance, CA, USA) with gradient elution, column temperature maintained at 40 °C, flow rate set to 300 μL/min, and injection volume fixed at 10 μL. Mobile phase A was methanol and mobile phase B was ethyl acetate, both supplemented with 0.1% formic acid. Mass spectrometric detection adopted electrospray ionization (ESI) source under dual positive and negative ion modes; the ion spray voltage was set to 5000 V, the declustering voltage at 80 V, and the ion source temperature kept at 500 °C. The mass scan range covered *m/z* 100–1250. Metabolite annotation followed standard microbial metabolomics workflows based on LC-MS/MS data. Compounds were tentatively identified based on accurate mass deviation (<5 ppm), MS/MS matching score (>70), retention time shift (<0.2 min), comparison with published literature, and matching against the spectral database provided with the LC–MS/MS platform [30]. The relative peak areas of the detected metabolites were used for relative abundance analysis. Differences in metabolite relative abundances between treatments were analyzed using one-way ANOVA followed by Duncan’s multiple range test, with *p* < 0.05 considered statistically significant.

### 2.4. In Planta Biocontrol Efficacy Evaluation

Seeds of *Brassica napus* cv. Huayouza 62 used in all pot and detached tissue trials were supplied by the College of Agriculture, Yangtze University. Greenhouse cultivation parameters were fixed at 25 °C, 16 h light/8 h dark photoperiod and 60% relative humidity. Seedlings at the 4–5 leaf stage were selected for all *in planta* tests. YZUS007 was cultured in LB broth for 48 h at 28 °C and 150 rpm. For the preparation of the cell-free filtrate, the bacterial culture was centrifuged at 10,000 rpm for 15 min at 4 °C, and the resulting supernatant was filtered through a 0.22 μm microporous membrane. Six experimental treatments were designed: CK (pathogen inoculation control), ZC (*S. sclerotiorum* + 20 μg/mL ZnO NPs), BC (*S. sclerotiorum* + YZUS007 bacterial culture), BZ (*S. sclerotiorum* + YZUS007 bacterial culture + 20 μg/mL ZnO NPs), EC (*S. sclerotiorum* + cell-free filtrate of YZUS007), and EZ (*S. sclerotiorum* + cell-free filtrate of YZUS007 + 20 μg/mL ZnO NPs). In the combined BZ and EZ treatments, ZnO NPs were adjusted to a final concentration of 20 μg/mL in the treatment solutions. All groups were uniformly inoculated with *S. sclerotiorum*, and the CK group served as the sole reference for calculating relative biocontrol efficacy. A non-inoculated water blank control was not included because the primary objective of this experiment was to compare disease suppression among pathogen-inoculated treatments under a uniform infection pressure. Therefore, all experimental groups were inoculated with *S. sclerotiorum*, and CK served as the reference for calculating relative biocontrol efficacy.

#### 2.4.1. Detached-Stem Assay

Healthy *B. napus* stems were cut into segments of 15 cm length, surface sterilized with 75% ethanol and rinsed three times with sterile distilled water. A 5-mm *S. sclerotiorum* mycelial plug was placed at the middle of each stem segment, followed by dropping 20 μL corresponding treatment solution onto the inoculation site. Lesion lengths were recorded at 72 h post-inoculation (hpi).

#### 2.4.2. Potted Plant Assay

A total volume of 10 mL treatment solution was evenly sprayed onto leaves of individual rapeseed seedlings. Each treatment contained three independent biological replicates with 10 plants per replicate. Prior to inoculation, *S. sclerotiorum* was cultured on PDA medium at 22 °C in darkness for 3 d to obtain uniform mycelial plates. Pathogen plugs with a diameter of 5 mm were punched from the actively growing edge of fungal colonies for inoculation. Seedlings were inoculated with a 5-mm pathogen plug on the third fully expanded leaf 24 h after spraying [31]. Disease evaluation adopted a 0–4 visual grading scale adapted from established disease assessment methods for *S. sclerotiorum* in rapeseed at 7 days post-inoculation (dpi): 0 = no lesions; 1 = lesion coverage below 5%; 2 = 5–25% leaf area damaged; 3 = 25–50% leaf area damaged; 4 = lesion area exceeding 50% [31]. Relevant indicators were calculated by the following formulas:

Incidence = (number of diseased leaves/total number of investigated leaves) × 100%

Disease index = ∑ (number of diseased leaves at all levels × disease progression)/(total number of surveyed leaves × highest disease progression) × 100

Relative control effect = (disease index of the control group − disease index of the treatment group)/disease index of the control group × 100%

### 2.5. Plant Defense Response Analysis

#### 2.5.1. Measurement of Defense Enzyme Activities

Leaf tissue samples for enzyme activity detection were collected at 12, 24, 48 and 72 hpi. Fresh leaf tissues were fully homogenized on ice using pre-cooled extraction buffer supplied by Solarbio assay kits. The homogenate was centrifuged at 12,000× *g* for 10 min at 4 °C, and the resulting supernatant was collected as crude enzyme solution. Commercial assay kits from Solarbio Science & Technology Co., Ltd. (Beijing, China) were adopted to determine the activities of superoxide dismutase (SOD), catalase (CAT), peroxidase (POD) and phenylalanine ammonia-lyase (PAL), following the manufacturer’s standard protocols strictly. All enzyme activity values were normalized to fresh leaf weight and expressed as U/g·FW. Each treatment contained three independent biological replicates, and each sample was measured with three technical replicates.

#### 2.5.2. Quantitative Real-Time PCR (qPCR) Analysis

Leaf tissues for RT-qPCR were sampled at 0, 12, 24, 48 and 72 hpi. Total RNA was extracted from leaf samples using the RNAprep Pure Plant Kit (Tiangen, Beijing, China). RNA concentration and purity were detected with a NanoDrop 2000 spectrophotometer (Thermo Fisher Scientific, Wilmington, DE, USA). Transcription levels of defense-related genes (*RBOH*, *PR1*, *WRKY33*) were quantified via one-step RT-qPCR with the UltraSYBR Kit (Vazyme Biotech Co., Ltd., Nanjing, China), using *Actin* as the internal reference gene. The forward and reverse primers for *RBOH*, *PR1*, *WRKY33*, and *Actin* were as follows: *RBOH*, forward 5′-CCATGCTCACGTCTGTTGCAA-3′ and reverse 5′-GTTTAGCGAAGTGGGACTTAACA-3′; *PR1*, forward 5′-AGACTCGTACACTCCGGTGG-3′ and reverse 5′-GTTGACCCAAAGGTTCACGG-3′; *WRKY33*, forward 5′-AACAACTACTGTGCCGGGT-3′ and reverse 5′-GAAAGAGGAAGACTCATCGCTG-3′; and *Actin*, forward 5′-GCAGACCGTGATGAGCAAAG-3′ and reverse 5′-GGAAGACGAAGATGGACAC-3′. Each sample was tested in three technical replicates. The 2^−ΔΔCT^ method was adopted to calculate relative gene expression levels, and all amplification tests were set in triplicate [32,33]. One-way ANOVA combined with Duncan’s multiple range test was applied to distinguish inter-group differences, with (*p* < 0.05) defined as the threshold for significant variation.

### 2.6. Statistical Analysis

All experimental data were processed and analyzed using SPSS 22.0 software (SPSS Inc., Chicago, IL, USA). One-way ANOVA was used to compare differences among treatment groups, and for measurements collected at multiple sampling time points, the analysis was performed separately at each time point. Duncan’s multiple range test was used for multiple comparisons. A value of *p* < 0.05 was considered statistically significant. Three biological replicates were included for each treatment, with three technical replicates for the corresponding measurements.

## 3. Results

### 3.1. Isolation, Identification and Antifungal Characterization of Strain YZUS007

**Figure 1:**
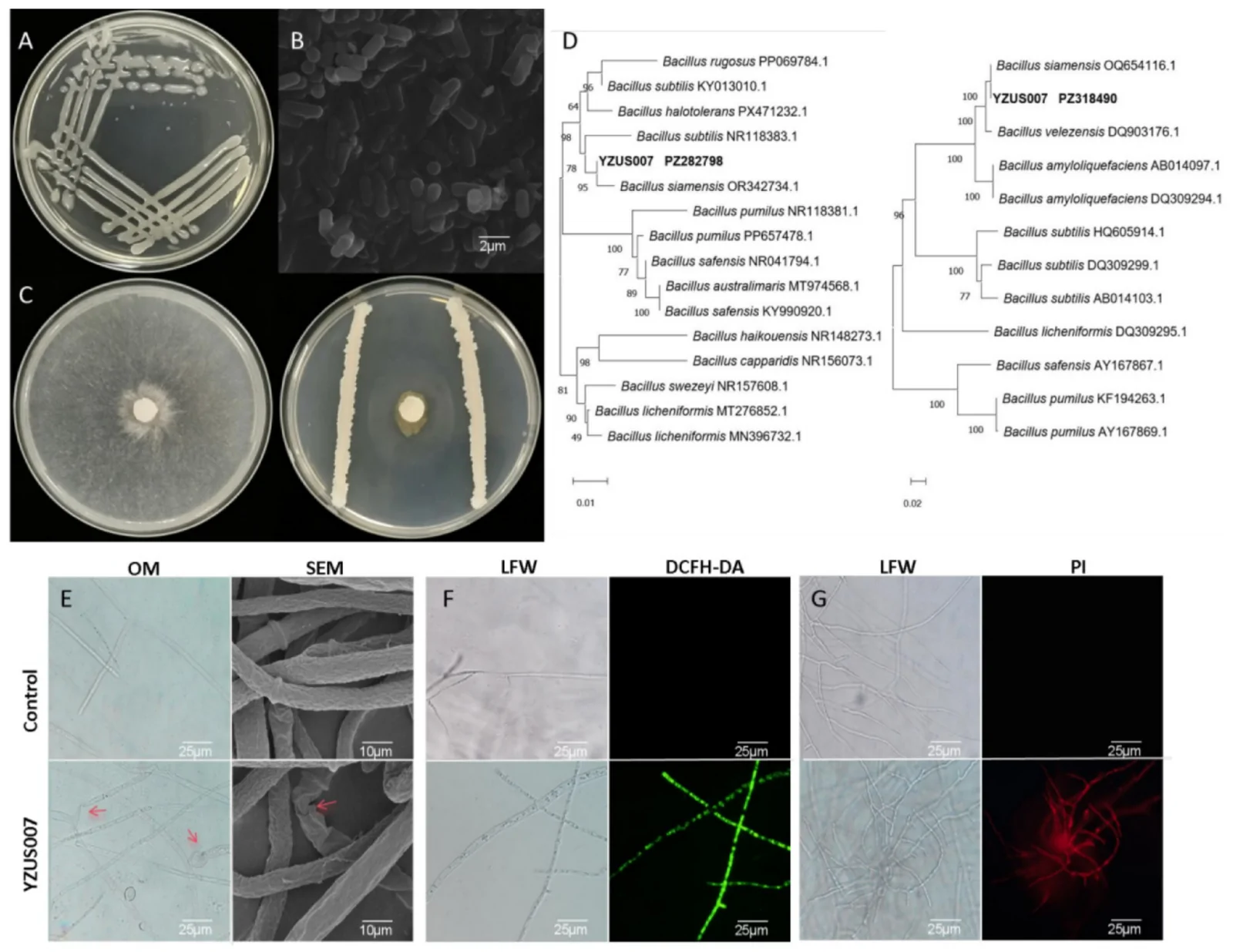
Isolation, Identification and Antifungal Characterization of Biocontrol Strain YZUS007. (**A**) Colony morphology of YZUS007 cultured on LB agar plate for 24 h. (**B**) Scanning electron microscopy (SEM) image of YZUS007 cells. (**C**) Dual culture assay showing the antagonistic effect of YZUS007 against *S. sclerotiorum*. Left panel: untreated *S. sclerotiorum* control; right panel: YZUS007 treatment group. (**D**) Neighbor-joining phylogenetic trees based on partial 16S rRNA and *gyrB* gene sequences. Bootstrap values (1000 replicates) are shown at branch nodes. (**E**) Optical microscopy (OM) and scanning electron microscopy (SEM) observation of *S. sclerotiorum* hyphae with or without YZUS007 treatment. OM images are displayed on the left side, and corresponding SEM images are placed on the right side. (**F**) ROS accumulation in *S. sclerotiorum* hyphae detected by DCFH-DA staining. (**G**) Propidium iodide (PI) Cell membrane integrity evaluated by PI staining.

A total of 126 bacterial isolates were recovered from rhizosphere soil attached to healthy *Brassica napus* roots, and dual-culture screening was carried out to select strains with inhibitory effects against *S. sclerotiorum*. Among all isolates, YZUS007 exhibited the strongest antagonistic capacity and was selected for subsequent functional verification. When incubated on LB agar for 24 h, this strain formed milky white, opaque colonies with irregular margins (Figure 1A). Scanning electron microscopy further confirmed its typical smooth rod-shaped cellular morphology (Figure 1B). A clear inhibitory zone formed between the bacterial streak and fungal colony, with an average inhibition rate of 78.4 ± 0.7% (Figure 1C).

Phylogenetic analyses were performed based on 16S rRNA and *gyrB* gene sequences, with phylogenetic trees constructed separately for each marker using the corresponding reference sequences. The phylogenetic trees showed that YZUS007 clustered tightly with reference strains of *Bacillus siamensis* supported by high bootstrap values on the main branches (Figure 1D). Its 16S rRNA and *gyrB* gene sequences have been deposited in GenBank under accession numbers PZ282798.1 and PZ318490, respectively. Based on sequence similarity to reference sequences in NCBI and phylogenetic analysis, YZUS007 was identified as *Bacillus siamensis.* Given the taxonomic complexity within the *Bacillus subtilis* species complex, this species assignment should be interpreted based on the sequence and phylogenetic evidence provided by the two markers used in this study.

Co-cultivation treatments induced severe structural damage to pathogen hyphae. Light microscopic observation revealed obvious swelling, wrinkling and distortion of hyphal filaments, while SEM imaging captured massive shrinkage and fragmentation of hyphal cell walls (Figure 1E). DCFH-DA staining displayed strong green fluorescence inside treated hyphae, indicating massive intracellular reactive oxygen species (ROS) accumulation (Figure 1F). PI staining generated intense red signals, demonstrating severe disruption of plasma membrane integrity (Figure 1G). All morphological evidence verified the robust antifungal potential of YZUS007 and justified its use as the core biocontrol strain in follow-up assays.

### 3.2. Physicochemical Characterization of ZnO NPs and Screening of a Suitable Working Concentration

**Figure 2:**
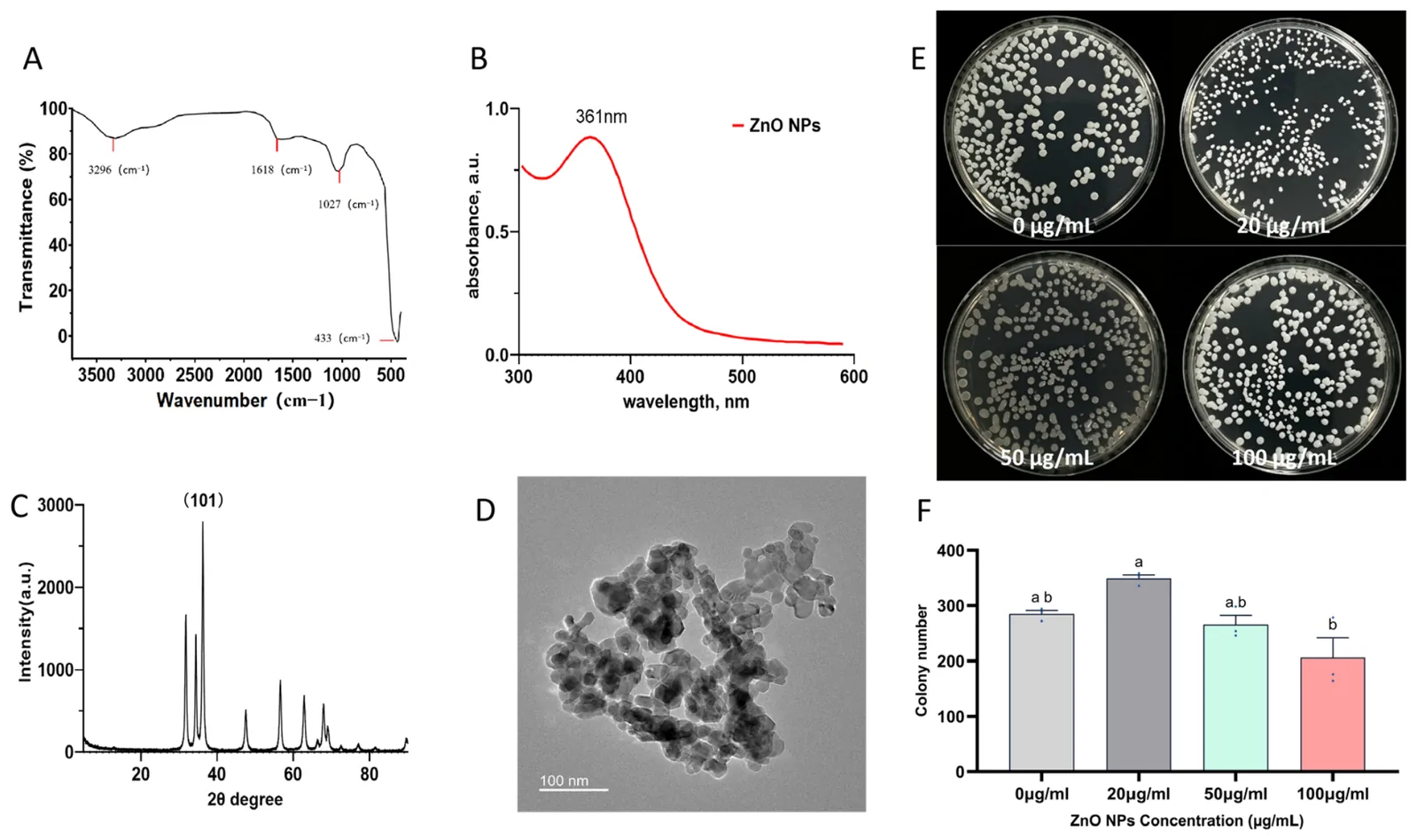
Physicochemical characterization of zinc oxide nanoparticles (ZnO NPs) and screening of a suitable working concentration for strain YZUS007. (**A**) Fourier-transform infrared (FTIR) spectrum of ZnO NPs. (**B**) Ultraviolet–visible (UV–Vis) absorption spectrum of ZnO NPs. (**C**) X-ray diffraction (XRD) pattern of ZnO NPs. (**D**) Transmission electron microscopy (TEM) image of ZnO NPs (scale bar = 100 nm). (**E**) Representative plates showing colony growth and colony morphology of YZUS007 under different concentrations of ZnO NPs. (**F**) Quantitative analysis of bacterial colony numbers under different ZnO NP treatments. Data are presented as the mean ± standard error (*n* = 3). Different lowercase letters indicate significant differences among treatments (*p* < 0.05).

Four complementary characterization techniques were applied to resolve the physicochemical properties of ZnO NPs. The FTIR spectrum presented a distinct absorption peak at 433 cm^−1^ assigned to Zn–O stretching vibration, which validated successful synthesis of zinc oxide crystals (Figure 2A). A characteristic absorption band at 361 nm was detected via UV–Vis spectroscopy, matching the optical signature of hexagonal wurtzite ZnO (Figure 2B). XRD peaks were consistent with the characteristic reflections of hexagonal wurtzite ZnO (JCPDS No. 89–1397) (Figure 2C). TEM micrographs illustrated nearly spherical nanoparticles with an average diameter of approximately 30 nm (Figure 2D).

Subsequent colony counting experiments evaluated the compatibility gradient of ZnO NPs with YZUS007. Plates supplemented with 20 μg/mL ZnO NPs exhibited higher colony-forming unit counts compared with the control, with visibly smaller individual colonies (Figure 2E,F). In contrast, higher concentrations (50 μg/mL and 100 μg/mL) reduced colony numbers. Under our testing conditions, 20 μg/mL ZnO NPs did not inhibit CFU formation of strain YZUS007 and was therefore chosen as our working concentration for subsequent assays.

### 3.3. In Vitro Antifungal Activity of YZUS007 and Targeted Metabolite Analysis

**Figure 3:**
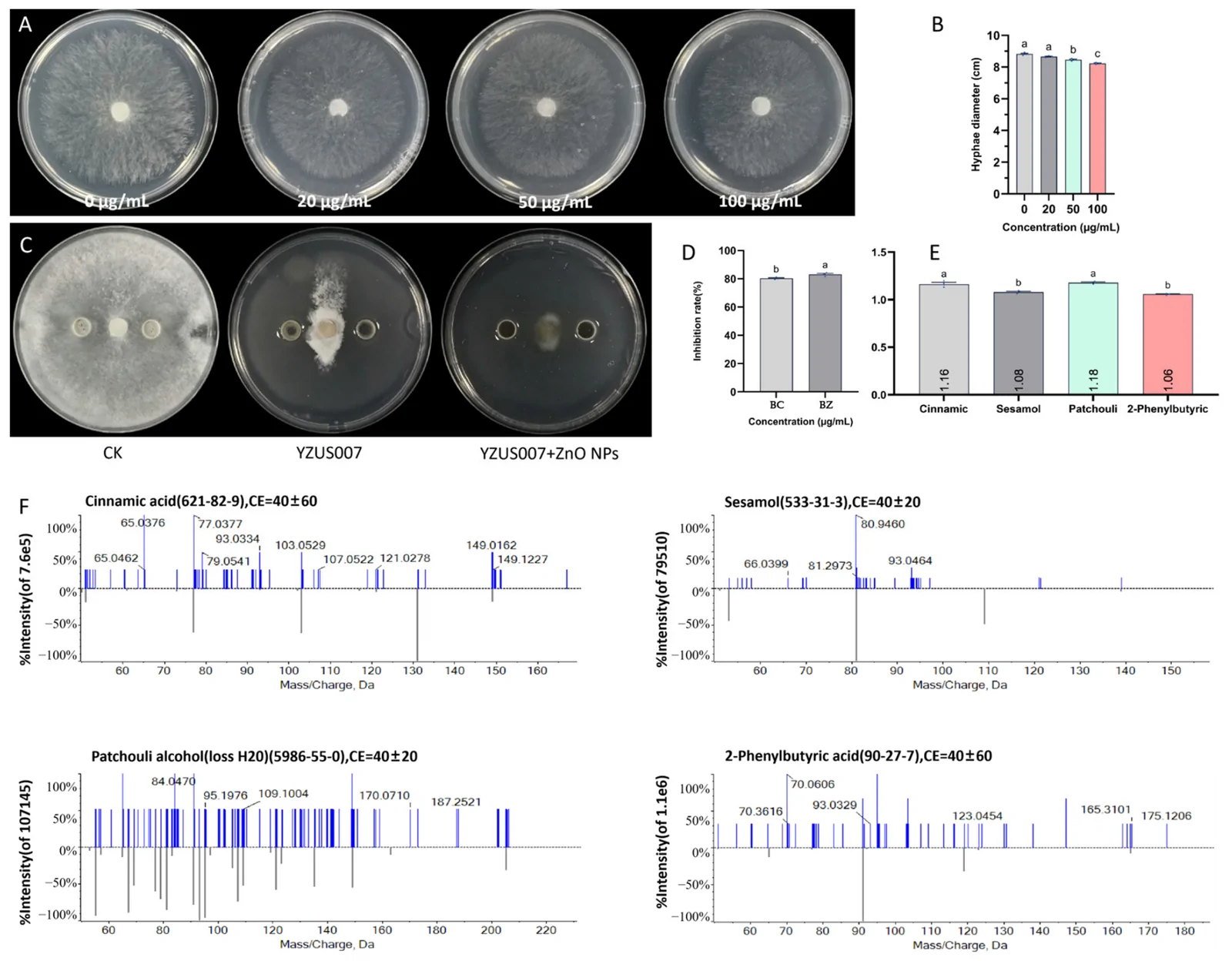
In vitro antifungal activity of ZnO NPs and enhancement of the antagonistic activity of YZUS007. (**A**) Representative plates showing mycelial growth of *S. sclerotiorum* under different concentrations of ZnO NPs. (**B**) Quantitative analysis of mycelial diameters of *S. sclerotiorum* treated with 0, 20, 50, and 100 μg/mL ZnO NPs. (**C**) Representative Oxford cup assay showing the antagonistic activity of the BC and BZ groups against *S. sclerotiorum*. (**D**) Quantitative analysis of inhibition rates in the BC and BZ groups. (**E**) Relative abundances of four metabolites identified by targeted LC–MS/MS analysis in the BC and BZ groups. (**F**) Representative MS/MS spectra of the four tentatively annotated metabolites identified by targeted LC–MS/MS analysis. Data are presented as the mean ± standard error (*n* = 3). Different lowercase letters indicate significant differences among treatments (*p* < 0.05). Notes: BC = *S. sclerotiorum* + YZUS007 bacterial culture; BZ = *S. sclerotiorum* + YZUS007 bacterial culture + 20 μg/mL ZnO NPs.

Single application of ZnO NPs produced negligible inhibitory effects on *S. sclerotiorum* mycelial expansion within the tested concentration range. Fungal colonies displayed highly similar morphology across all ZnO NP concentration gradients (Figure 3A). Quantified mycelial diameter only decreased marginally from 8.83 cm (control) to 8.23 cm at the maximum tested dosage of 100 μg/mL (Figure 3B). No statistically significant difference was detected between the blank control and the 20 μg/mL ZnO NP group, indicating that this concentration did not significantly inhibit the growth of the target pathogen under the tested conditions. This concentration was therefore selected to test whether ZnO NPs could amplify the strain’s intrinsic antagonistic performance.

Oxford cup assays were conducted to evaluate the enhanced in vitro antifungal capacity associated with 20 μg/mL ZnO NPs. Both BC and BZ groups generated clear, measurable inhibition zones against *S. sclerotiorum* (Figure 3C). Statistical quantification revealed that the inhibition rate rose significantly from 80.27% (BC group) to 83.07% (BZ group) (Figure 3D). Such data indicated that low-dose ZnO NPs moderately strengthened the strain’s in vitro antagonistic capacity.

Targeted LC–MS/MS metabolomics was performed to uncover shifts in extracellular secretory profiles induced by ZnO NP supplementation. Among the detected metabolites, four compounds—cinnamic acid, sesamol, patchouli alcohol and 2-phenylbutyric acid—showed significantly different relative abundances between the BC and BZ groups (*p* < 0.05) and were tentatively annotated (Figure 3E,F). Compared with the BC group, all four compounds exhibited higher relative abundances in the BZ group, with fold changes ranging from 1.06 to 1.18 (Figure 3E). These metabolomic data show that low-concentration ZnO NPs were associated with changes in the relative abundance of extracellular metabolites in *Bacillus siamensis* YZUS007.

### 3.4. Biocontrol Efficacy Against Sclerotinia Stem Rot in Planta

**Figure 4:**
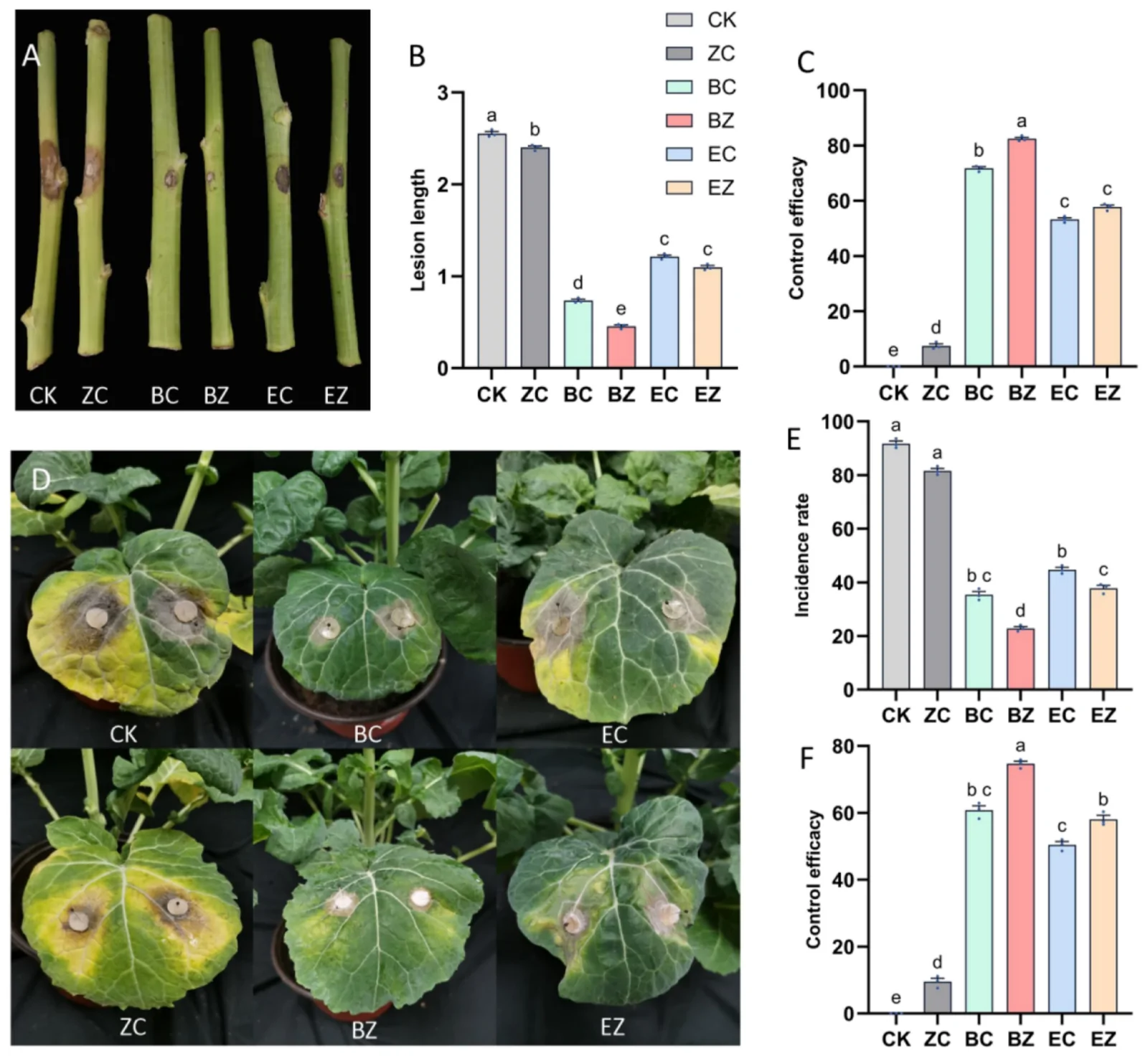
Biocontrol efficacy of different treatments against Sclerotinia stem rot of *Brassica napus* under detached-stem and potted plant conditions. (**A**) Disease symptoms of detached *B. napus* stems at 72 h post-inoculation (hpi). (**B**) Lesion lengths of detached *B. napus* stems under different treatments. (**C**) Biocontrol efficacies of different treatments in the detached-stem assay. (**D**) Disease symptoms of potted *B. napus* leaves at 72 hpi. (**E**) Disease incidence rates of potted *B. napus* leaves under different treatments. (**F**) Biocontrol efficacies of different treatments in the potted plant assay. Data are presented as the mean ± standard error (*n* = 3). Different lowercase letters indicate significant differences among treatments (*p* < 0.05). Notes: Six experimental treatments were set in this assay: CK = pathogen inoculation control; ZC = *S. sclerotiorum* + 20 μg/mL ZnO NPs; BC = *S. sclerotiorum* + YZUS007 bacterial culture; BZ = *S. sclerotiorum* + YZUS007 bacterial culture + 20 μg/mL ZnO NPs; EC = *S. sclerotiorum* + cell-free filtrate of YZUS007; EZ = *S. sclerotiorum* + cell-free filtrate of YZUS007 + 20 μg/mL ZnO NPs.

Detached-stem and potted seedling assays were jointly adopted to evaluate the *in planta* disease-suppressing performance of all six experimental treatments. In the detached-stem trial sampled at 72 hpi, the lesion length of the CK pathogen-inoculated control reached 2.55 cm. Single ZnO NP treatment (ZC) significantly reduced lesion length to 2.40 cm compared with the CK group, although the absolute difference was small. Both BC and EC treatments markedly suppressed lesion development, achieving relative control efficiencies of 71.73% and 53.26%, respectively (Figure 4B,C). Combined BZ treatment further elevated control efficacy up to 82.45%, which was significantly higher than the BC single-strain group. The EZ group also generated moderately improved control efficiency (57.74%) relative to EC, though the inter-group gap did not reach statistical significance.

Consistent changing trends were reproduced in potted plant inoculation experiments. Disease incidence was 91.72% in the CK group and 81.61% in the ZC group (Figure 4E,F). BC and EC treatments both greatly alleviated disease severity, delivering 60.79% and 50.44% relative control efficacy separately. The BZ combined treatment achieved the maximum protective effect at 74.68%, significantly surpassing BC alone. Meanwhile, EZ treatment exhibited a 58.08% control rate, which was statistically superior to the EC cell-free filtrate group.

### 3.5. Induction of Plant Defense Responses

**Figure 5:**
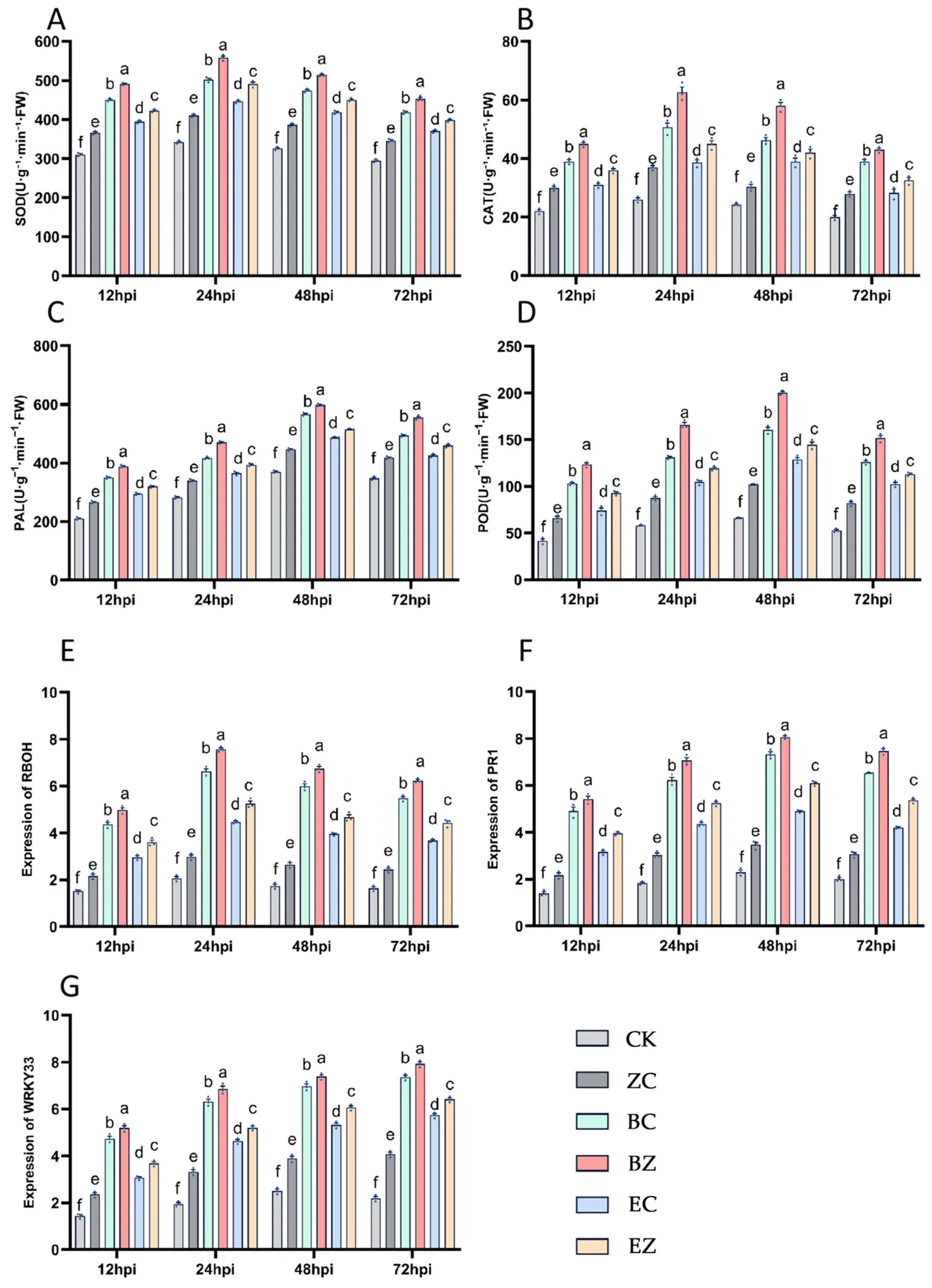
Effects of different treatments on defense enzyme activities and defense-related gene expression in *Brassica napus* leaves. (**A**) SOD activity; (**B**) CAT activity; (**C**) PAL activity; (**D**) POD activity; (**E**) *RBOH*; (**F**) *PR1*; (**G**) *WRKY33*. Data are presented as mean ± standard error (*n* = 3). Different lowercase letters above bars indicate significant differences among treatments at the same time point at *p* < 0.05. Notes: Treatment abbreviations are consistent with those defined in Figure 4.

Dynamic variations in four defensive enzyme activities and three defense-associated gene transcripts were measured at 12, 24, 48 and 72 hpi to interpret the molecular basis of amplified *in planta* biocontrol efficiency. Activities of SOD, CAT, POD and PAL differed among treatments at every sampling time point. All biocontrol treatments triggered far higher enzyme activity than CK and ZC groups, with an overall trend of initial elevation followed by gradual decline. The BZ combined treatment consistently maintained the highest enzyme activity levels across all time gradients. SOD and CAT activity peaked at 24 hpi, whereas PAL and POD reached maximum activity at 48 hpi (Figure 5A–D). At their respective peak time points, enzyme activities of the BZ group were significantly higher than those measured in the BC group. The EZ group also generated elevated enzyme activity compared with EC, yet the magnitude of improvement remained weaker than the gap between BZ and BC.

Identical dynamic patterns were observed for the transcription of defensive marker genes *RBOH*, *PR1* and *WRKY33* (Figure 5E–G). All biocontrol treatments induced significant upregulation of these three genes relative to CK and ZC controls. Transcript abundance of *RBOH* peaked at 24 hpi, while *PR1* and *WRKY33* reached maximum expression at 48 hpi and 72 hpi respectively. Among all six treatments, BZ constantly exhibited the highest gene expression levels. Transcript accumulation in EZ was also higher than EC, though the degree of upregulation remained inferior to the contrast between BZ and BC.

Overall, defense enzyme activities and defense-related gene expression in BZ were approximately 1.5-fold and 1.8-fold those in BC, respectively. Compared with EC, the corresponding responses in EZ were approximately 1.3-fold and 1.5-fold those in EC, respectively, while those in ZC were approximately 1.2-fold and 1.3-fold those in CK, respectively.

## 4. Discussion

Biocontrol agents based on *Bacillus* strains have been widely acknowledged as eco-friendly alternatives to chemical fungicides for managing sclerotinia stem rot disease [34]. In the present work, rhizosphere-isolated *Bacillus siamensis* YZUS007 exerted potent antagonistic effects against *S. sclerotiorum* and triggered severe structural damage to fungal hyphae. These observations align with well-documented characteristics of *Bacillus* biocontrol strains, whose suppressive capacity arises from secretion of antimicrobial metabolites and disruption of pathogen cellular integrity.

We have a key observation that supplementation with 20 μg/mL low-dose ZnO NPs significantly enhanced the antagonistic performance of YZUS007. Distinct from most prior reports that adopted high ZnO NP dosages (50–200 μg/mL) to achieve direct antifungal effects [35,36], the 20 μg concentration selected herein showed only negligible inhibition of *S. sclerotiorum* when applied alone. Previous publications have described biphasic dose responses of ZnO NPs toward microorganisms: low concentrations stimulate metabolic activity, while high doses restrain bacterial proliferation and biofilm formation [26,37]. Additional evidence reveals that ZnO NP concentrations exceeding approximately 25 ppm impose adverse impacts on the growth and biofilm construction of *Bacillus* species [38,39]. The 20 μg/mL dosage used in this trial sits below this reported threshold, consistent with the absence of growth inhibition observed for strain YZUS007. Collectively, these data indicate that optimizing the functional capacity of beneficial bacteria may provide an alternative strategy for disease suppression without increasing nanomaterial application doses.

LC-MS/MS metabolomic profiling indicated that ZnO NP exposure altered the extracellular secretory profile of YZUS007 and was associated with higher relative abundances of four metabolites: cinnamic acid, sesamol, patchouli alcohol and 2-phenylbutyric acid. All four compounds belong to bioactive aromatic secondary metabolites with well-documented broad-spectrum antifungal and antibacterial capacities in previous phytochemistry and microbial metabolism studies [40]. Cinnamic acid exerts prominent antifungal activity and imposes stronger growth-inhibitory effects on fungal pathogens than many other aromatic acids by disrupting pathogen intracellular redox homeostasis and interfering with reactive oxygen species balance [41]; sesamol acts as a natural antioxidant that eliminates excess reactive oxygen radicals and thereby delivers auxiliary antimicrobial effects [42]; patchouli alcohol possesses multiple physiological regulatory activities, and its broad-spectrum antimicrobial potential against pathogenic microorganisms has been partially validated in existing research [43]; compounds with a 2-phenylbutyric acid skeleton show significant inhibitory activity against various plant pathogenic bacteria, confirming the inherent antibacterial bioactivity of this chemical scaffold [44]. Collectively, all four metabolites have independently documented antimicrobial bioactivity. It is reasonably speculated that their increased relative abundances may contribute to the enhanced in vitro antagonistic performance observed in the BZ treatment group. Further targeted functional testing is still required to quantify the individual disease-suppressing contribution of each metabolite against *S. sclerotiorum*. Whether and how these metabolites may additionally contribute to plant defense responses beyond their direct antimicrobial effects remains to be determined.

Notably, the enhanced disease-suppressing effect detected in intact plant tissues far exceeded the mild improvement observed under in vitro culture conditions. Both detached-stem and potted seedling assays displayed stronger control efficiency for the combined BZ treatment relative to the single bacterial inoculation, whereas cell-free filtrate supplemented with ZnO NPs only generated limited efficacy gains. This discrepancy implies that secreted metabolites alone cannot fully account for the elevated biocontrol performance. Prior mechanistic research has demonstrated that metal oxide nanoparticles can adsorb and stabilize small bioactive molecules via electrostatic surface interactions, delaying their degradation and extending functional durability [45]. This adsorption effect may contribute to the slight efficacy improvement seen in the EZ filtrate group. Nevertheless, the substantially stronger synergism observed with live YZUS007 cells suggests complex tripartite interactions among ZnO NPs, biocontrol bacteria and host plant tissues may contribute to the enhanced disease resistance.

Subsequent analysis of plant defensive responses further validated the involvement of host immune activation in the synergistic mechanism. Co-treatment with YZUS007 and ZnO NPs significantly elevated the enzymatic activities of SOD, CAT, POD and PAL, alongside upregulated transcription of *RBOH*, *PR1* and *WRKY33*. The *RBOH* gene governs early reactive oxygen burst during plant immune signaling, while *WRKY33* acts as a master transcription factor regulating host resistance against necrotrophic fungi [46,47,48]. *PR1* serves as a canonical molecular marker of plant defense activation [49]. These results suggest that ZnO NPs do not merely strengthen direct bacterial antagonism against pathogens; they also enhance the strain-associated activation of plant defense responses. Numerous studies have proven that *Bacillus* strains activate plant immunity through microbe-associated molecular patterns (MAMPs), yet whether ZnO NPs modulate this signal transduction process remains undetermined [50]. The current dataset cannot determine the relative contributions of modified metabolite secretion and altered plant–microbe signal exchange to the elevated defense responses. All three pathways may work in tandem to produce the amplified defensive phenotype recorded in BZ-treated plants.

Rather than acting as a standalone fungicide, low-concentration ZnO NPs function as microbial growth and function boosters to enhance the biocontrol potential of *Bacillus* YZUS007. The superior disease suppression of the combined treatment may involve two coordinated pathways: strengthened bacterial antagonism and amplified host defensive activation [23]. Multiple ecological studies have documented adaptive tolerance responses of environmental microorganisms following long-term nanomaterial exposure, highlighting the value of minimizing unnecessary nanoparticle input in agricultural ecosystems [51]. Compared to application schemes relying on high ZnO NP concentrations for direct pathogen suppression, the low-dose synergistic strategy proposed in this work cuts nanomaterial usage while retaining reliable disease control. Since this approach primarily augments beneficial microbial activity instead of imposing strong selective pressure on pathogen populations, it may slow the evolution of fungicide-resistant variants, though long-term field trials are necessary to verify this inference. Future investigations should resolve the molecular signaling cascades mediating ZnO–bacterium crosstalk, assess field-scale control efficiency, and evaluate the long-term environmental safety of this composite biocontrol system.

## 5. Conclusions

The present study isolated and taxonomically identified *Bacillus siamensis* YZUS007 as an effective biocontrol strain capable of restraining *S. sclerotiorum*. Supplementation with ZnO NPs at 20 μg/mL significantly boosted the strain’s disease-suppressing performance under both in vitro culture and intact plant experimental systems. LC-MS/MS metabolomic detection indicated that ZnO NP supplementation was associated with changes in the relative abundances of YZUS007 extracellular metabolites. Assays measuring defensive enzyme activity and defense gene transcription further revealed that the combined treatment elicited stronger plant immune activation compared with single-strain application. Collectively, the enhanced biocontrol efficacy of the BZ group may be associated with both increased bacterial antifungal metabolism and intensified host defensive responses. This research provides solid experimental evidence supporting the application of low-dose ZnO NPs as microbial activity enhancers, instead of direct antimicrobial agents, and delivers a sustainable technical framework for the integrated management of rapeseed sclerotinia stem rot. Follow-up research should quantify the individual contribution of each identified antifungal metabolite and dissect the core molecular regulatory networks governing interactions among ZnO NPs, *Bacillus siamensis* and *Brassica napus* host plants.

## Data Availability

The original contributions presented in this study are included in the article. Further inquiries can be directed to the corresponding author.
